# SeniorSentry: Correlation and Mutual Information-Based Contextual Anomaly Detection for Aging in Place

**DOI:** 10.3390/s23156752

**Published:** 2023-07-28

**Authors:** Achyuth Nandikotkur, Issa Traore, Mohammad Mamun

**Affiliations:** 1 Deptartment of Electrical and Computer Engineering, University of Victoria, Victoria, BC V8P 5C2, Canada; itraore@ece.uvic.ca; 2 National Research Council Canada, Government of Canada, Ottawa, ON K1A 0R6, Canada; mohammad.mamun@nrc-cnrc.gc.ca

**Keywords:** IoT security, IoT sensors, aging in place, anomaly detection, contextual anomaly

## Abstract

With the ever-growing reliance on IoT-enabled sensors to age in place, a need arises to protect them from malicious actors and detect malfunctions. In an IoT smart home, it is reasonable to hypothesize that sensors near one another can exhibit linear or nonlinear correlations. If substantiated, this property can be beneficial for constructing relationship trends between the sensors and, consequently, detecting attacks or other anomalies by measuring the deviation of their readings against these trends. In this work, we confirm the presence of correlations between co-located sensors by statistically analyzing two public smart-home datasets and a dataset we collected from our experimental setup. Additionally, we leverage the sliding window approach and supervised machine learning to develop a contextual-anomaly-detection model. This model reaches a true positive rate of 89.47% and a false positive rate of 0%. Our work not only substantiates the correlations but also introduces a novel anomaly-detection technique to enhance security in IoT smart homes.

## 1. Introduction

Aging in place refers to the idea of older adults maintaining their independence as they age by living in their own homes and communities without having to move to a different living environment, such as an assisted living facility or a nursing home. Technology can significantly support this goal by providing useful IoT systems and solutions [1]. However, with the growing reliance on such systems, it is crucial to ensure that they operate optimally and that any anomalies or deviations that may suggest a problem are detected early to prevent negative consequences. Especially in smart homes designed for older adults, the detection of anomalies becomes critical and we classify these anomalies into two types:

1. Intentional anomalies: They refer to deviations from normal behavior that are created deliberately, rather than occurring naturally or by chance. These anomalies are created by malicious actors with the aim of disrupting the normal functioning of the system or causing harm. Tampering with sensors, performing network attacks and spoofing are all examples of methods that can create intentional anomalies.
2. Unintentional anomalies: They refer to deviations from normal behavior that are not intentionally created, but rather occur due to a variety of factors, such as sensor malfunctions, environmental interference, user error, improper installation and power fluctuations.

Whether the anomalies are created intentionally or unintentionally, their impact on seniors is the same. For example, a malicious actor may intentionally launch a denial-of-service attack on the smart home’s lighting system, causing the lights to shut off and thus increasing the danger of falls or other accidents for aging residents. Similarly, if the motion-detection sensor in the home malfunctions and fails to detect movement, the lights may not turn on, resulting in a similar adverse situation. Overall, the importance of anomaly detection in smart homes designed for the elderly cannot be overstated. It is essential to therefore protect both the physical and emotional safety of these individuals and ensure that they can enjoy the benefits of smart-home technology seamlessly. A simple method of anomaly detection is to establish a region that represents normal behaviour and identify any observation that falls outside of this region as an anomaly. However, several factors make this approach challenging, as noted by [2], such as the difficulty of capturing the concept of normalcy. In this study, we propose a novel technique that models the relationships among sensors to detect intentional and unintentional anomalies. By modelling these relationships, it is possible to establish normal patterns for sensor readings and thus identify anomalies that deviate from these patterns.

### 1.1. Contextual Anomaly Detection

Cook et al. [3] presented an alternate categorization for anomalies in time series IoT data, dividing them into three types: point anomaly, contextual anomaly and collective anomaly. Contextual anomaly is defined as an observation that may otherwise appear to be normal, but when considered within a context, can be considered an anomaly. To construct a contextual-anomaly-detection model, we first need to learn or identify the contextual and behavioural attributes of the system. Contextual attributes are characteristics of the environment or context in which the sensor data is collected. For instance, in multivariate time series datasets, time can serve as a contextual attribute. Behavioural attributes are characteristics of the data that indicate its behaviour or pattern over time. One common behavioural attribute in time-series data is the trend, which refers to the general direction or pattern of the data over time. Contextual anomalies can be particularly challenging to detect, as they only appear abnormal within a specific context. For example, a sudden increase in the number of steps taken by an older person in the middle of the night, as recorded via a wearable fitness tracker, could indicate an anomaly. The high number of steps in itself is not an anomaly; however, in the context of the time of the day, it is considered an anomaly. Therefore, there is a need to discover various contexts and apply them to observations to establish normal vs abnormal activity.

Hayes et al. [4] employed the idea of profiles to create contexts by grouping similar data points together using a multivariate clustering algorithm, and applied it to detect contextual anomalies. Carmona et al. [5] proposed an anomaly-detection framework, called neural-contextual-anomaly detection (NCAD) which incorporates contexts through a contextual hypersphere. In this study, we aim to use the relationships among sensors as contexts for anomaly detection. Co-located sensors often exhibit linear or nonlinear relationships [6]. Using these relationships as behavioral attributes and a sliding window of a fixed number of samples as a contextual attribute for contextual anomaly detection has not been explored, to the best of our knowledge, and our study aims to fill this gap. Although correlation can measure linear relationships, it is not useful for representing a nonlinear relationship between two random variables. This is where mutual information can be useful. Mutual information measures the amount of information shared between two variables. It quantifies the amount of reduction in uncertainty about one variable due to given knowledge of the other variable and it can be used to identify nonlinear relationships between variables. Ultimately, our objective is to identify anomalies, whether intentional or unintentional, that violate the linear and nonlinear correlations among sensors using correlation and mutual-information scores.

### 1.2. Our Work

In this study, we first prove the presence of linear or nonlinear relationships among co-located sensors in a smart home by performing correlation analysis on two public benchmark datasets, the open smart-home dataset [7] and the smart-building dataset [8], and a dataset we collected in our laboratory at the University of Victoria, called the ISOT AgeTech dataset. Second, we use a sliding window of a fixed number of samples (i.e., the contextual attribute) on the ISOT AgeTech dataset to determine the correlation and mutual-information scores between sensor readings (i.e., behavioral attributes). Thirdly, we use these measurements as features to train selected machine-learning models and evaluate their ability to detect anomalies. We apply the proposed method (i.e., the second and third steps) solely to the ISOT AgeTech dataset and not to the other datasets mentioned because it is the only dataset with benign and anomalous samples. Therefore, the public benchmark datasets are used only to substantiate the existence of correlations between sensors.

### 1.3. Paper Organization

The remainder of this paper is organized as follows. Section 2 presents related work. Section 3 describes the different datasets used in our exploratory study and presents the results of correlation analysis performed on those datasets. Section 4 describes normal and anomalous data in the context of this work. Section 5 presents our detection model and underlying data analysis. Section 6 describes the experimental evaluation and obtained results. Finally, Section 7 concludes the paper and also summarizes our future work.

## 2. Related Work

Several statistical techniques for detecting outliers have been proposed in the existing literature [9,10,11]. Apart from the statistical techniques, there also exist density [12], distance [13], clustering [14] and machine learning-based anomaly-detection techniques [15].

However, a key problem with most existing anomaly-detection methods is that they tend to ignore the context in which the data is generated. This lack of consideration of context can lead to incomplete and inaccurate results [4]. Consideration of the context is particularly crucial for detecting anomalies in smart homes designed for older people.

Artola et al. [16] recognized this lack of research on contextual anomaly detection in relation to the well-being and healthcare of older adults. They proposed a system that uses a wearable device to collect data on older adults’ heart rate, sleep duration and daily step count (i.e., behavioural attributes) in relation to the time of day (i.e., contextual attribute). Then, they utilized multiple open-source anomaly-detection models to identify any deviations and report them to a healthcare provider.

Shahid et al. [17] focused on building a model to learn behavioural patterns of older adults in smart homes and proposed an anomaly-detection model that learns behavioural attributes, namely the amount of time spent and number of visits the resident makes to each room of the house (i.e., contextual attribute). The researchers then applied a non-parametric statistical method based on Chebyshev’s inequality theorem to detect anomalies in daily user activities. They defined an outlier as an observation whose duration exceeded what was expected by two standard deviations. This method is limited by the use of Chebyshev’s inequality, in which thresholds are based on loose intervals and 75% of the data fall within two standard deviations. This is less precise than a normally distributed dataset, in which 95% of the data fall within two standard deviations. Having wider intervals between thresholds can result in fewer anomalies because fewer data points fall outside the defined range.

Aran et al. [18] collected data from pressure, motion and door sensors located in 40 different households of elderly people to model their daily behaviour and identify anomalies. They developed a probabilistic spatio-temporal model to summarize normal behaviour and used cross-entropy measures to identify and categorize significant deviations from the norm as anomalies. However, the proposed approach suffers from the unavailability of ground-truth labels and the inability to generalize to multiple residents.

In the broader context of IoT smart homes, Chenglong et al. [19] introduced a semantics-aware anomaly-detection system termed the Home Automation Watcher (HAWatcher). This system models the normal behavior in smart homes by generating correlations from semantic information, such as installation locations, device types, smart apps, configurations and relations. These correlations are categorized into two types: e2e (event to event) and e2s (event to state). The HAWatcher also contains a shadow execution engine that simulates the states of various devices based on the observed correlations. Any deviation between the simulated and real-world device states is flagged as an anomaly. Overall, it achieved an impressive precision of 97.83% and a recall of 94.12%, notably surpassing previous methods. However, since a discrete set of state transitions are considered in the context of observed correlations, only a limited type of anomalies can be detected.

Researchers so far have focused on modelling the behavior of older adults, using relevant AgeTech sensors, to perform context-based anomaly detection. However, only a few focused on modelling the relationship between such sensors to do the same. While we could not find any research that specifically uses the relationship between AgeTech sensors for contextual anomaly detection, we did find a few studies that make limited use of these relationships in a different domain.

For example, Deng et al. [6] used a graph structure learning approach to determine the relationship between the sensors associated with water treatment and water distribution. They also employed a graph attention-based forecasting method to predict the expected value of a sensor at a specific time and classified observations as anomalies if the deviation between the forecasted and actual values exceeded a threshold. However, this technique relies on prediction and does not fully consider the continuous nature of the correlation and mutual information between the sensors.

Li et al. [20] proposed a method that involves creating a temporal correlation graph by analyzing the correlation between different features in an industrial multi-sensor system and then using a specialized neural network (called a structured-sensitive graph neural network) to extract useful information from the graph, such as the relationships between points, edges and overall structure. This information, combined with preset thresholds on the fluctuations of correlation and sensor values, is then utilized to classify the graph and detect any anomalies. Although this technique models the linear relationship between the sensors using correlation coefficients, it cannot model nonlinear relationships between sensors.

Current methods of context-based anomaly detection usually rely on the pre-identification off both contextual and behavioural attributes, with the assumption that the context is determined by spatial or temporal characteristics. However, in reality, it can be difficult to identify the true context in a dataset, particularly when the dataset is high-dimensional and has numerous attributes that can be combined in different ways to create the context [21]. No prior methods exist, to our knowledge, that take into account both the linear and nonlinear relationships between AgeTech-related IoT sensors to build an anomaly-detection model.

## 3. Datasets

### 3.1. AgeTech Sensors and Data

Two major perspectives can be emphasized when collecting AgeTech datasets: infrastructure and activity. The infrastructure perspective defines the environment in which a senior person lives, while activity captures their daily routines. Although these perspectives are interdependent, one can be emphasized over the other in data collection. Similarly, our proposed detection model focuses primarily on the infrastructure aspects. A combination of different sensor types can be deployed in a typical AgeTech environment. Table 1 provides several examples of common sensors that can be found in AgeTech smart homes.

After an extensive search, we could not find publicly available IoT-sensor datasets related to aging in place. As an alternative, we utilized the open smart home, smart building and ISOT AgeTech datasets to substantiate the existence of correlations between co-located sensors in a smart home. Subsequently, we used the ISOT AgeTech dataset solely to validate our model, as only it has both benign and anomalous samples.

In the following sections, you will find an overview of these three datasets.

### 3.2. Open Smart-Home Dataset

The open smart-home dataset was collected at Fraunhofer Institute for Building Physics, Nürnberg, Germany by Schneider et al. [7]. It contains time series measurements of temperature, brightness and humidity sensors placed in the bathroom, kitchen, rooms 1, 2 and 3 and the toilet of a smart home located in this building. The placement of the various sensors is shown in Table 2 and the corresponding counts of the data samples pertaining to each sensor are shown in Table 3.

### 3.3. Smart-Building Dataset

The smart-building dataset was collected in Sutardja Dai Hall (SDH) at UC Berkeley by Hong et al. [8]. It contains time series measurements from 255 sensors that were placed across 51 rooms. Each room had a CO_2_-concentration sensor, an air-humidity-measurement sensor, an air-temperature-measurement sensor, a luminance sensor and a passive-infrared-ray (PIR) sensor. The distribution of data samples per sensor type is listed in Table 4.

The timestamps of the collected data samples were in Unix Epoch Time. All sensors were sampled once every 5 s, while the PIR motion sensor was sampled once every 10 s. The PIR sensor helps determine the presence of a person inside a room by measuring the radiation emitted from the subjects in its proximity.

### 3.4. ISOT AgeTech Dataset

Temperature, humidity and air-quality sensors are commonly used in homes designed for older adults to monitor the indoor environment and ensure that it remains safe and comfortable for residents. In addition, they are readily available and highly dependable, which is why they were utilized in collecting the ISOT AgeTech dataset. The dataset contains sensor data collected from two DHT22 and two MQ135 sensors located in the ISOT laboratory at the University of Victoria. Each DHT22 sensor contains one built-in temperature sensor and one built-in humidity sensor. The descriptions of the DHT22 and MQ135 sensors are given in Table 5.

#### Collection Network Architecture

**Figure 1:**
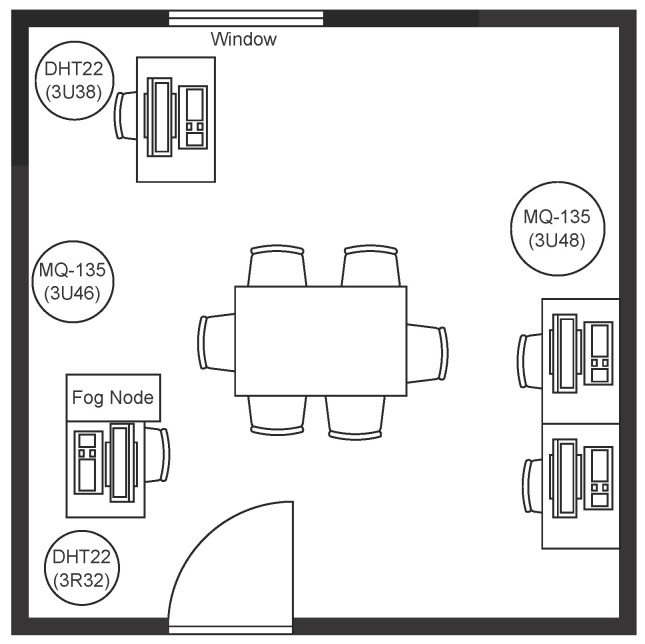
Floor plan and placement of the sensors in the ISOT laboratory.

The floor plan of the laboratory and placement of the sensors is shown in Figure 1. The collection network architecture consists of three components: IoT sensors, a fog node and a cloud server. The fog node is responsible for aggregating information from different IoT sensors over a certain time and sending it to the cloud server. Sensor information is posted by the corresponding micro-controller unit (ESP32) to the fog node using HTTP REST protocol and the cloud server provides data storage and eventually data-processing capability (e.g., using machine-learning models). In total, we collected data from the following six sensors:

1. Two temperature sensors (DHT22).
2. Two humidity sensors (DHT22).
3. Two air-quality-measurement sensors (MQ135).

**Figure 2:**
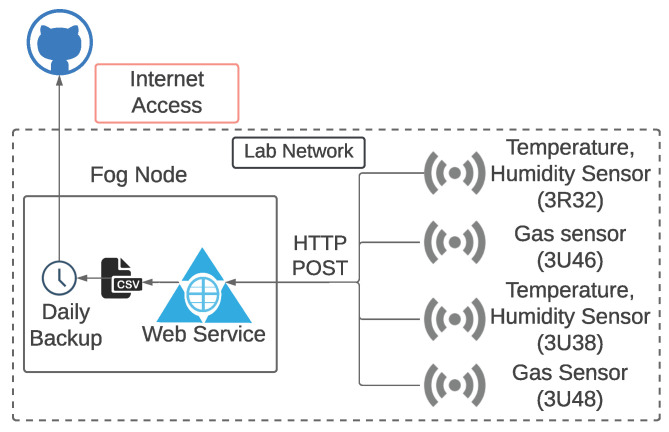
ISOT laboratory setup.

Each sensor sends its data every 30 s to the Web API hosted on the fog node (a machine in the ISOT laboratory). A new file is created for each such sensor and uploaded to our GitHub repository once every day [22], as shown in Figure 2. The datasets starting with keywords 3R32 and 3U38 contain the temperature and humidity information collected from the two DHT22 sensors, whereas the datasets starting with keywords 3U46 and 3U48 contain the air-quality information sourced from the two MQ135 sensors. The top five rows of the dataset pertaining to the temperature and humidity sensor (3R32) are listed in Table 6.

## 4. Exploring Normal and Anomalous Data

Both the open smart-home and smart-building datasets consist solely of normal data. The sensors and procedures used to collect this data are outlined in Section 3.2 and Section 3.3. In contrast, the ISOT AgeTech dataset includes both normal and anomalous samples, which are detailed in the following sections.

### 4.1. Normal Data of ISOT AgeTech Dataset

The normal samples in the ISOT AgeTech dataset represent observations captured during regular human activity in the experiment room of the ISOT laboratory. During the capture period, five individuals worked in the room from 9 AM to 8 PM and the room remained empty overnight. All sensors were placed next to the individual carrels and there was constant human traffic in and out, which was captured by the sensors. It is well known that human presence can affect the ambient temperature, humidity [23] and CO_2_ [24] in enclosed spaces.

### 4.2. Anomalous Data of ISOT AgeTech Dataset

As explained in Section 1, in the context of aging-in-place smart homes, anomalies can be categorized into two types: intentional and unintentional. Their roles in the ISOT AgeTech dataset are presented below:

*Intentional Anomalies:* As a conduit to produce intentional anomalies, we conducted two types of network-based attacks on the web service hosted on the fog node.

1. Distributed denial-of-service (DDOS) attacks: These attacks were conducted to disrupt the collection of sensor readings and effectively disable the fog node. To achieve this, we used an open-source tool called the PYbot Botnet [25].
2. Replay attacks: These attacks were executed by altering the payloads and replaying the previously captured HTTP requests sent by various sensors to the fog node.

A malicious actor can choose to use randomly generated sensor data during these attacks, but this is a simplistic and unsophisticated method that can be quickly identified via our model, as randomly generated sensor data readily violates sensor relationships. A more natural approach is to use data that resembles sensor data. Therefore, we chose to source these sensor values from the attack data of another IoT-sensor dataset called the TON_IoT weather dataset [26,27,28,29,30,31,32] which was generated from the same type of sensors as the ISOT AgeTech dataset. It is important to note that our model does not necessarily detect attacks but the anomalous conditions that these types of network attacks may create, which violate the correlation between sensors.

*Unintentional Anomalies:* These anomalies are typically caused by sensor malfunctions, incorrect installation by the user, improper usage or environmental interference. To simulate such anomalies, we altered the sensitivity of the MQ135 sensors by intermittently adjusting the potentiometers to their maximum or minimum values and periodically disconnecting the wiring for the DHT22 sensors. This resulted in abnormal sensor values that can serve as representative examples of unintentional anomalies. Gaddam et al. [33] referred to such anomalies as intermittent sensor errors and binary failures. Because the data from various sensors reside in different files, we first merged all of the files collected between 4 September 2022 and 7 September 2022. All the sensor readings within the range of 30 s were merged into a single timestamp. Table 7 shows the top five rows of the merged dataset. At a particular time, if one or more sensors are anomalous, the entire row would be labelled an anomaly. Table 8 shows the breakdown of normal and anomalous samples in the merged dataset. The full dataset can be accessed at [34].

## 5. Proposed Detection Model

**Figure 3:**
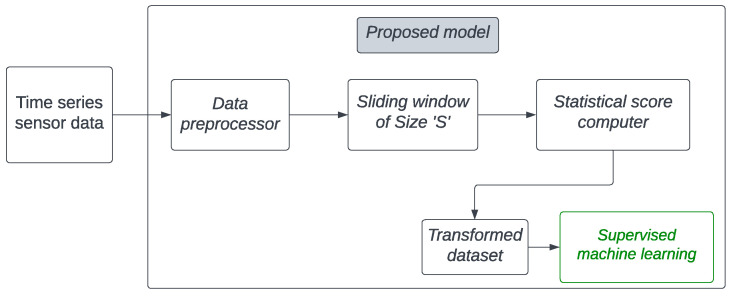
Components of the proposed method.

The proposed detection model utilizes correlation and mutual-information scores between the values of various co-located sensors as features (i.e., behavioural attributes). Additionally, a sliding window of a fixed number of samples (i.e., the contextual attribute) is employed to extract these features. For classification, we trained three different machine-learning models and evaluated their ability to detect contextual anomalies in sensor data. As illustrated in Figure 3, the proposed model processes the data through a series of components outlined below, before employing supervised machine learning for classification.

- Data preprocessor: This component merges the values from various sensors against a single timestamp using a tolerance of *T* seconds. Additionally, it removes rows containing any missing values.
- Sliding window: This provides a context within which statistical scores are calculated.
- Statistical score computer: This component calculates the correlation and mutual-information scores between sensors over *S* rows within each window, creating a single new row in the transformed dataset. Consequently, each row in the transformed dataset represents the statistical relationship between sensor values across *S* rows of the original dataset.
- Supervised machine learning: Machine-learning classification models are subsequently trained with the transformed dataset to develop an anomaly-detection model.

### 5.1. Exploratory Data Analysis

**Figure 4:**
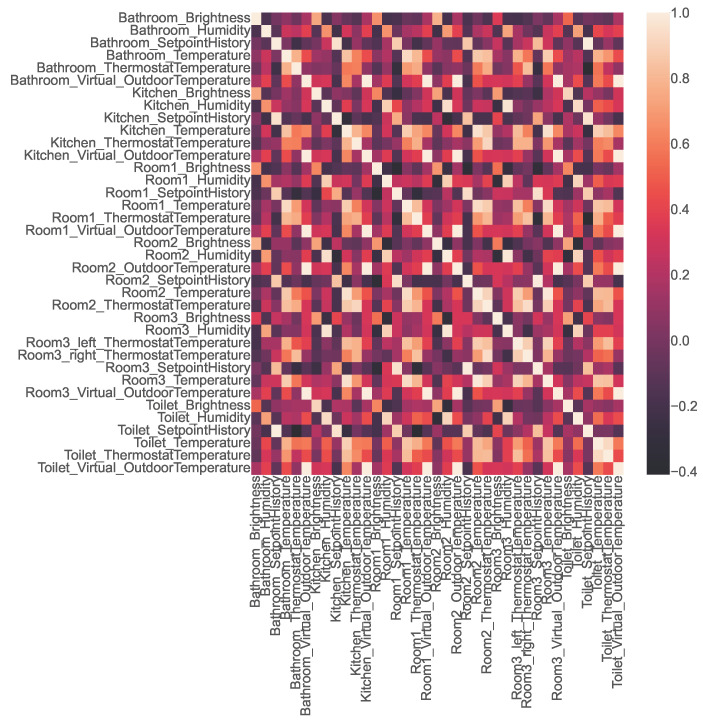
Correlation heatmap representing the correlations among the sensors in the open smart-home dataset.

The open smart-home dataset contains readings from 25 physical sensors positioned in various rooms of the smart home, as shown in Table 2. It also features data from six virtual sensors that capture the air temperature outside each room as obtained from a virtual weather service. To test whether the values of any of these co-located sensors were correlated, we used Pearson’s correlation coefficient to construct the correlation matrix shown in Figure 4. The lighter blocks in the matrix indicate a positive correlation, while the darker blocks indicate a negative correlation. Valuable insights can be drawn from the heatmap by examining the strength of these correlations.

**Figure 5:**
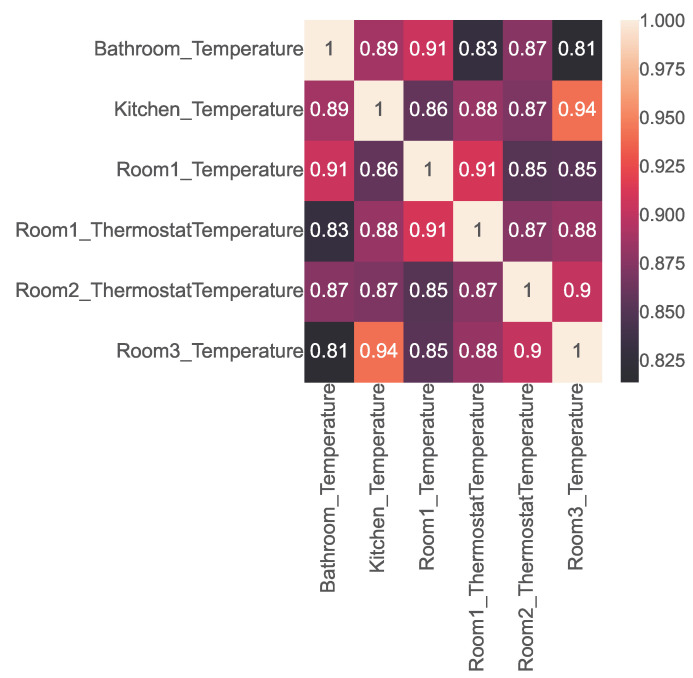
Correlation heatmap representing the correlation among 30 very strongly positively correlated sensor pairs in the open smart-home dataset.

As mentioned by [35] and shown in Table 9, two variables are very strongly positively correlated if the coefficient value is between 0.8 and 1. Similarly, two variables are very strongly negatively correlated if the coefficient value is between −1 and −0.8. We used this reference range to extract the sensor pairs that were very strongly positively or negatively correlated and obtained the following results:

1. A total of 85 sensor pairs were found to be very strongly positively correlated; 30 of them are shown in Figure 5.
2. No strong negatively correlated sensors were found.

**Figure 6:**
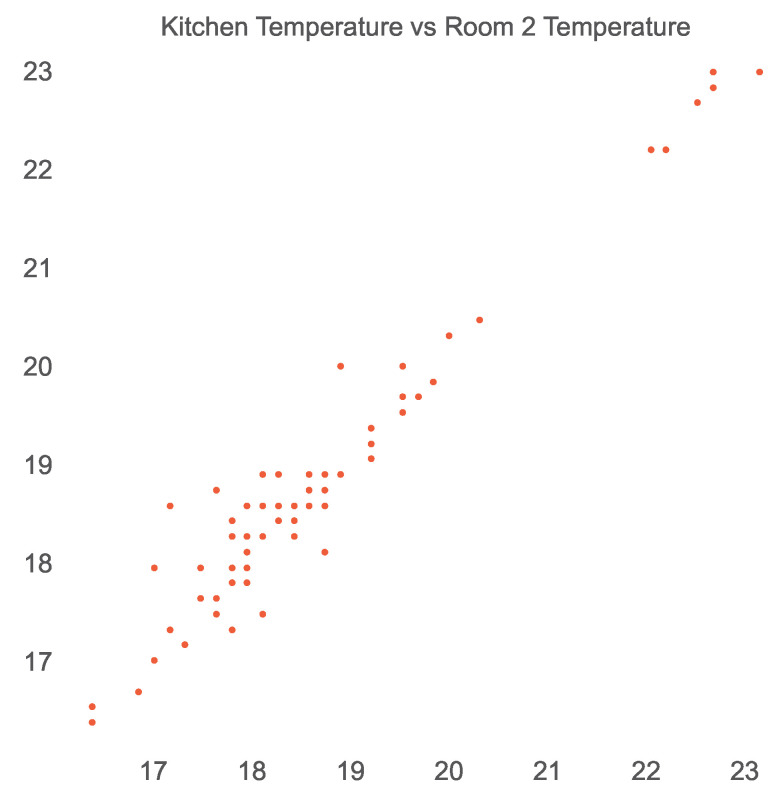
Linear relationship among temperature sensors in the kitchen and room 2 of the open smart-home dataset.

**Figure 7:**
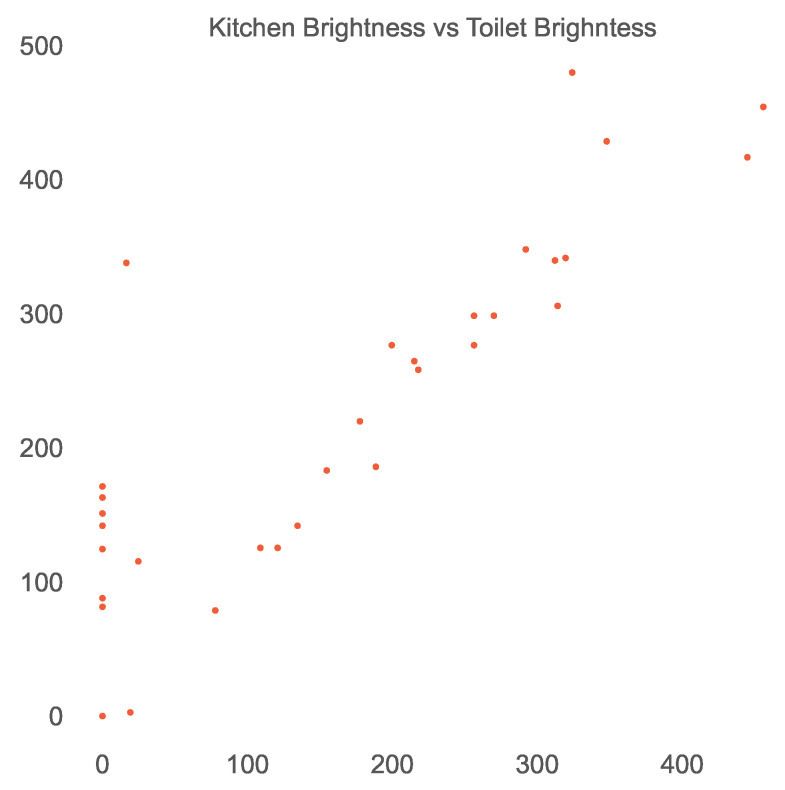
Linear relationship of brightness sensors in the kitchen and toilet of the open smart-home dataset.

The scatter plot shown in Figure 6 reveals the linear relationship between the temperature sensors in the kitchen and room 2. Similarly, a linear relationship between the brightness sensors in the kitchen and toilet can be seen in Figure 7. Thus, we established the existence of a spatial and temporal correlation between some co-located sensors in a smart home. It is also interesting to note that these relationships may also indicate a physical connection between different rooms in a smart home. For example, the correlation between brightness or temperature sensors in the kitchen and room 2 may also suggest that the kitchen and room 2 are physically connected. However, this idea is yet to be investigated.

**Figure 8:**
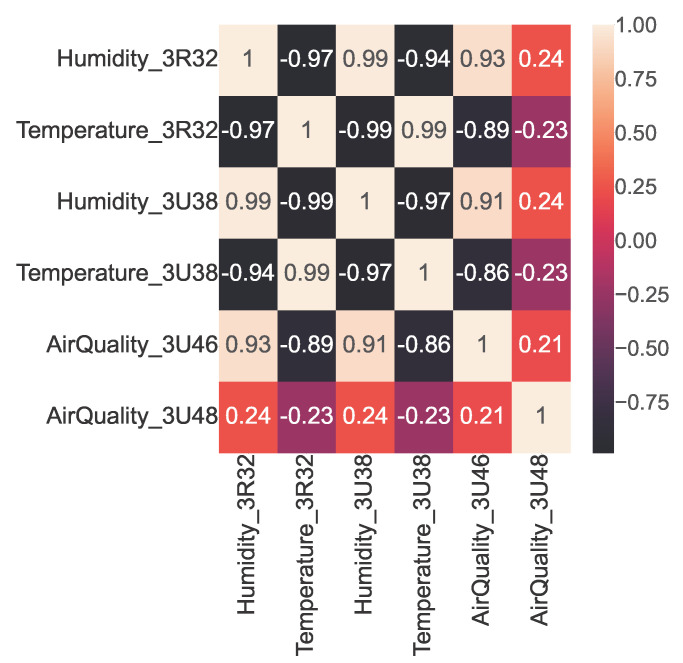
Correlation heatmap among six sensors in the ISOT dataset.

**Figure 9:**
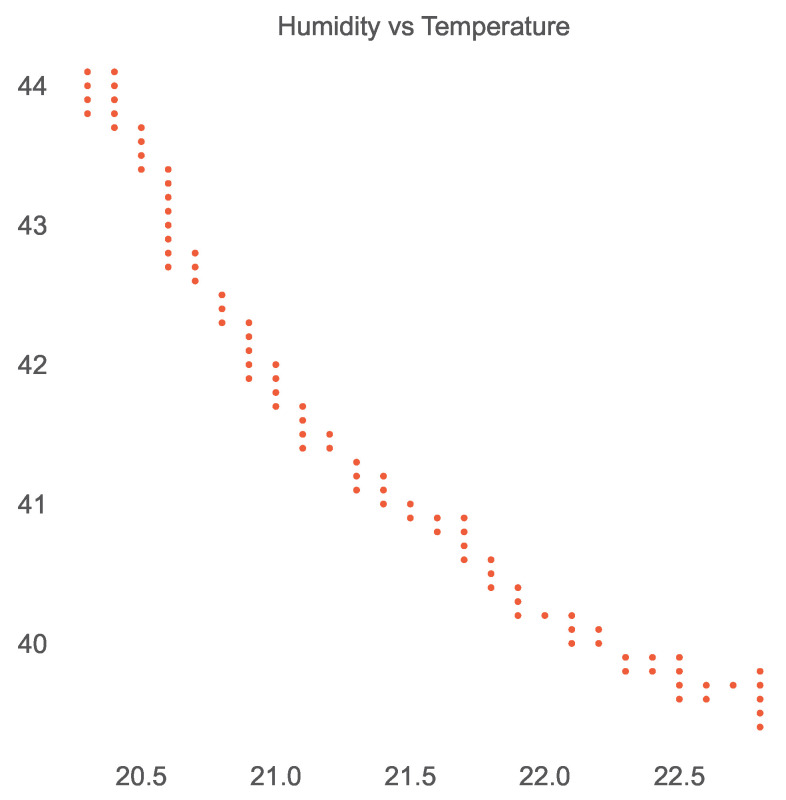
Negative correlation of temperature and humidity sensors in the ISOT AgeTech dataset.

**Figure 10:**
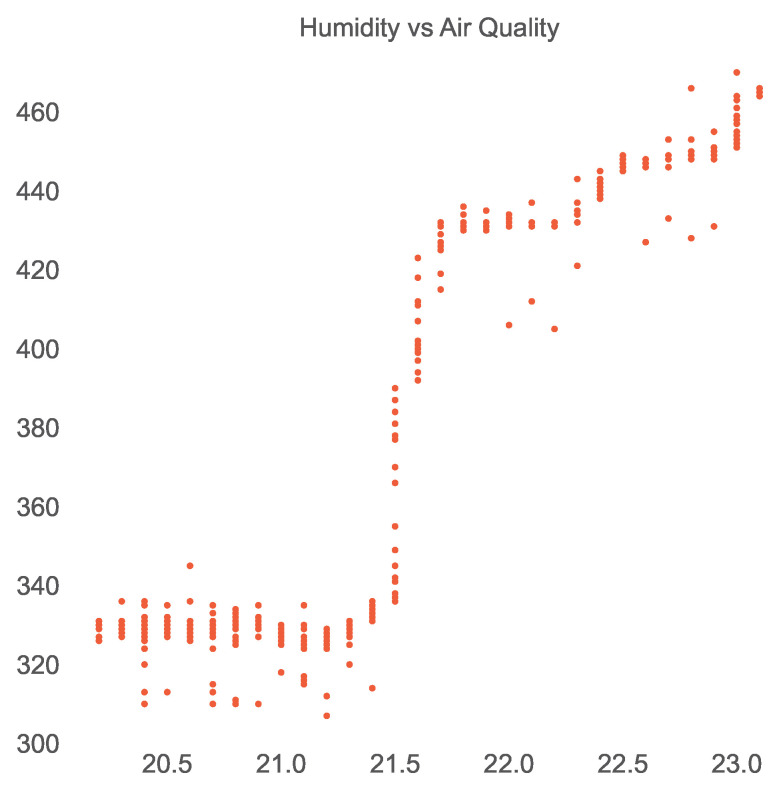
Nonlinear relationship between humidity and air-quality sensors in the ISOT AgeTech dataset.

Similar to the open smart-home dataset, we used Pearson’s correlation coefficient to identify the strength of linear dependencies among the sensors of the ISOT AgeTech dataset and produced the corresponding correlation heatmap shown in Figure 8. We then extracted the very strongly positively or negatively correlated sensor pairs using Pearson’s coefficient interpretation, presented in Table 9. We found that the temperature and humidity sensors were correlated negatively as shown in Figure 9 and humidity and air-quality sensors were found to maintain a nonlinear relationship as shown in Figure 10.

**Figure 11:**
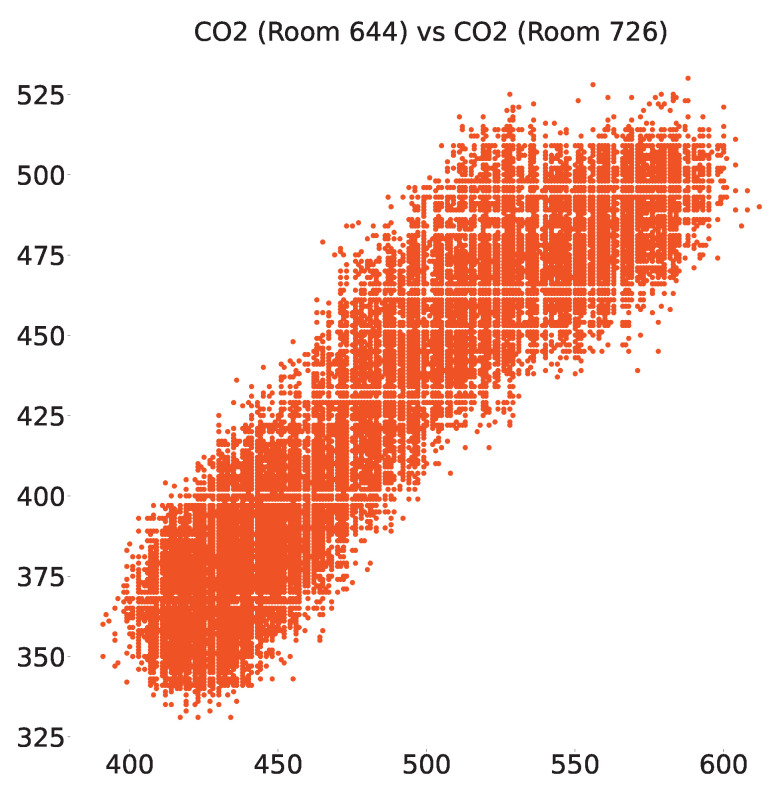
Positive correlation of temperature sensors in rooms 717 and 721 of the smart-building dataset.

**Figure 12:**
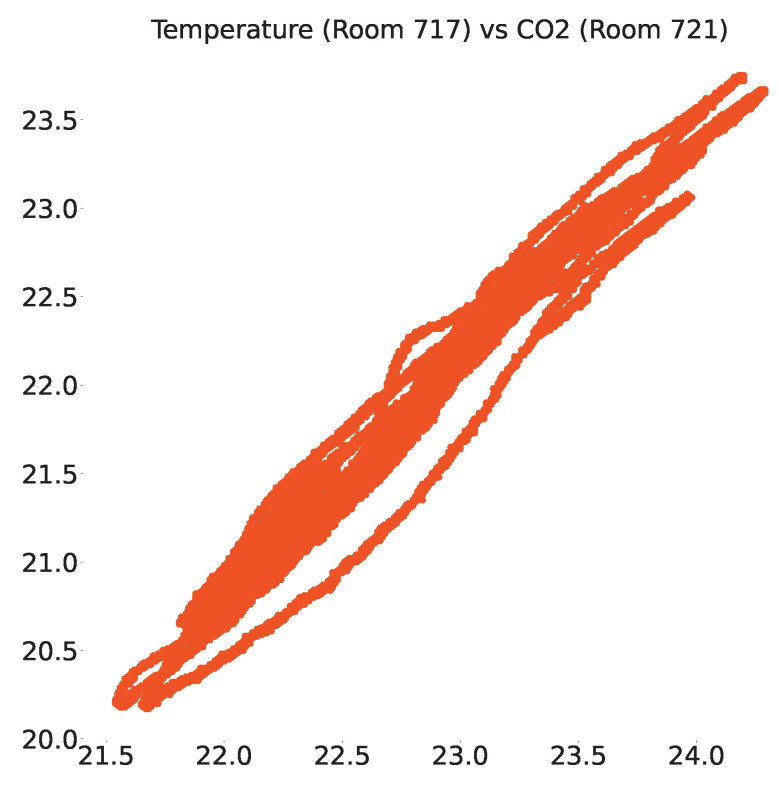
Positive correlation between CO_2_ sensors in rooms 644 and 726 of the smart-building dataset.

The same analysis was performed on the smart-building dataset and we found a total of 67 sensors that were positively correlated very strongly, out of which the scatter plot between CO_2_ sensors in rooms 644 and 726 and temperature sensors in rooms 717 and 721 are shown in Figure 11 and Figure 12, respectively.

### 5.2. Feature Model

One of the key concepts behind the proposed technique is to create new features that capture the relationships between sensors in an IoT-sensor dataset, from the existing features that represent sensor values.

**Figure 13:**
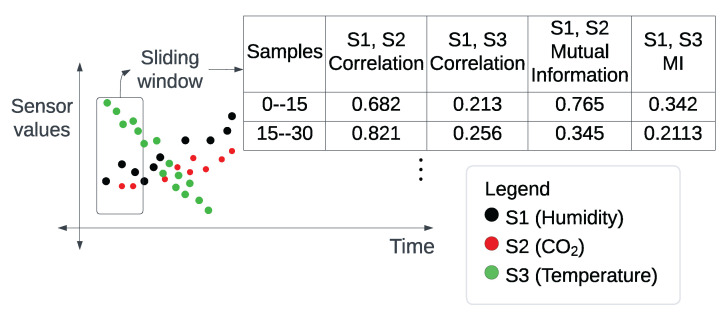
Sliding window to extract correlation and mutual information values.

To explain this in more detail, let us consider the example of the ISOT AgeTech dataset and its features. From the raw sensor data of this dataset, using the sliding window approach shown in Figure 13, we extracted a total of 30 features by calculating the correlation and mutual-information scores between the sensor pairs. In this manner, one sample with these new features was created for each of the old (or raw) samples that fall within the range of the sliding window. If the range contained any samples with a type that was not benign, the newly created sample was labelled as anomalous.

### 5.3. Classification Models

Using our proposed feature model, we explored three different machine-learning classifiers: random forest, naive bayes and k-nearest neighbours (k-NN).

Random Forest: Random forest is a machine-learning algorithm used for classification and regression tasks. It is an ensemble method, meaning that it combines the predictions of multiple models to make a final prediction. In a random forest, a large number of decision trees are trained and their predictions are combined through majority voting. Each decision tree is trained on a randomly selected subset of the data and the final prediction is made by taking the majority vote (in classification tasks) or the average (in regression tasks) of the predictions made by the individual decision trees.

K-nearest neighbours algorithm: The k-NN algorithm is a simple, non-parametric method used for classification and regression. It is an instance-based learning algorithm, meaning that it does not learn a model from the training data but instead stores training data and makes predictions based on similarities in new data points to the stored training data. In the k-NN algorithm, a new data point is classified or regressed based on the majority class or average value of its k nearest neighbors, where k is a positive integer that is specified by the user. The nearest neighbours are determined based on a distance measure, such as Euclidean distance or Manhattan distance.

Naive Bayes classifier: Naive Bayes classifier is a probabilistic algorithm that uses Bayes’ theorem to classify data points. Bayes’ theorem describes the probability of an event occurring based on prior knowledge of conditions that might be related to the event. In the case of a Naive Bayes classifier, the event we are interested in is a class label and the prior knowledge is represented by the probabilities of certain features (also known as predictors or attributes) being associated with each class.

## 6. Experimental Evaluation

### 6.1. Evaluation Metrics and Procedure

Out of several evaluation metrics commonly used to evaluate the performance of anomaly-detection models, we chose the following set:

1. Accuracy: This is the proportion of observations that were correctly classified as either benign or anomalous. Accuracy is calculated as the ratio of the number of correct predictions to the total number of predictions.
2. True Positive Rate (TPR): This is the proportion of anomalous observations correctly identified by a model as anomalous. It is calculated as the ratio of the number of true positives (i.e., anomalous observations that are correctly classified as anomalous) to the total number of actual anomalous observations.
3. False Positive Rate (FPR): This is the proportion of benign observations that are incorrectly classified by a model as anomalous. It is calculated as the ratio of the number of false positives (i.e., benign observations that are classified as anomalous) to the total number of benign instances.
4. AUC-ROC: The area under the receive operating characteristic curve (AUC-ROC) is a commonly used evaluation metric. The ROC curve shows the relationship between a model’s TPR and FPR at different thresholds. AUC-ROC is a value between 0 and 1, which represents the overall performance of the model. A value of 1 indicates that the model has perfect performance and can perfectly distinguish between benign and anomalous instances, whereas a value of 0.5 indicates that the model’s performance is no better than random guessing.

For the experimental evaluation, we split the transformed ISOT AgeTech dataset into an 80–20 ratio to train the chosen machine-learning models. This was in line with the findings of [36], who indicated that the optimal results are obtained by using 20–30% of the data for testing and the remaining 70–80% for training. Additionally, we also computed the evaluation metrics with sliding windows of 15, 25 and 35 samples to determine the influence of window size on performance.

### 6.2. Evaluation Results

In the following, we present the performance results obtained using the three different classifiers considered in our study on the ISOT AgeTech dataset.

We used the sliding-window technique to transform this dataset into a new set containing correlation and mutual-information scores. These scores were then used as features to train the selected machine-learning classifiers. Stratified K-fold with K = 10 was used for cross-validation, while grid search was employed for hyperparameter tuning. Further, the results from applying these classifiers at different window sizes (15, 25 and 35) are shown in Table 10, Table 11 and Table 12.

The obtained results are very encouraging, particularly with the best results obtained for k-NN and a sliding window size of 25, which yielded high accuracy, AUC, TPR and low FPR. This underscores the strength of the proposed approach when the dataset includes linear or nonlinear relationships between the sensors. Furthermore, to compare the proposed method to traditional deep-learning approaches, we applied the LSTM (Long Short-Term Memory) and the Simple Recurrent Neural Network models to the ISOT AgeTech dataset. The results are presented in Table 13. These findings suggest that in scenarios where anomalies closely resemble normal data, the proposed method consistently outperforms traditional deep-learning techniques.

#### Discussion

Our analysis of the open smart-home, smart-building and ISOT AgeTech datasets, as presented in Section 5.1, revealed the presence of correlations among co-located sensors. This phenomenon can be attributed to various reasons:

- Shared environment: When sensors are co-located, they often share a similar environment. This means that external factors such as temperature fluctuations, humidity or light exposure will impact them simultaneously. For example, if a human subject enters a room, there will be a simultaneous change in the readings of many sensors such as temperature, humidity, CO2, PIR (passive infrared) and more.
- Measurement of related phenomena: Some sensors, though distinct, measure phenomena that are inherently related. A classic example is that of temperature and humidity. As the air’s temperature increases, if no additional moisture is added to the air, the relative humidity will decrease. Thus, a change in one could lead to a change in the other.
- Sensor Interference: In some cases, the operation or output of one sensor can influence the readings of another sensor nearby. This is especially true if sensors operate on similar frequencies or if one emits a signal (like infrared) that another sensor can pick up.

This finding allowed us to model relationships between sensors using statistical scores and utilize supervised machine learning for classification. Interestingly, these relationships sometimes might also suggest a physical connection between different rooms in a smart home, presenting a promising avenue for future research. The detection model outlined in Section 5 was designed to detect sensor readings that deviate from the typical correlation and mutual-information patterns seen so far in normal data. It is important to note that these relationships among sensors are not always present. In such scenarios, there will not be any correlation or mutual information patterns between the time-series readings of different sensors; as a result, our model will not be efficient in detecting anomalies.

**Figure 14:**
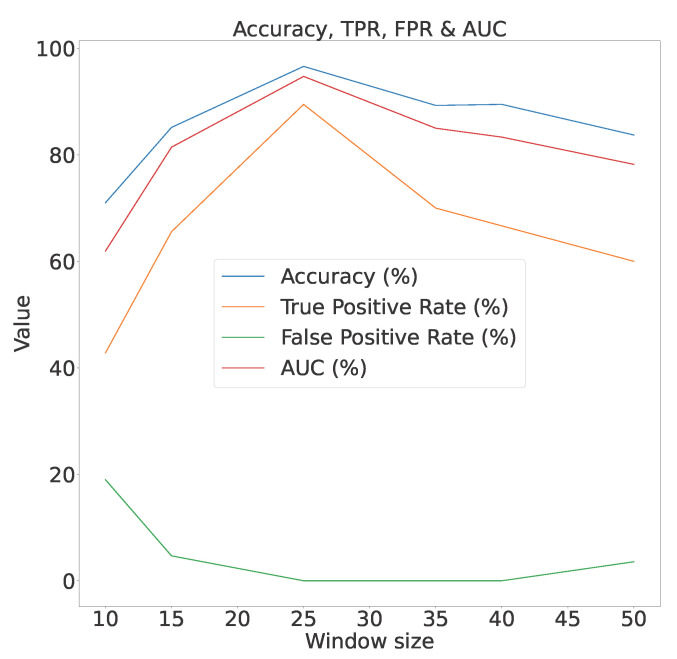
Accuracy, TPR, FPR and AUC vs window size of k-NN algorithm on ISOT AgeTech dataset.

Additionally, as the window size increases on the ISOT AgeTech dataset, we observed an improvement in accuracy and TPR, along with a reduction in the FPR. However, this trend eventually reached a critical point beyond which the accuracy and TPR declined and the FPR increased. This could be due to the increased availability of samples under a window, which in turn causes the correlation and mutual-information scores to become more representative of the underlying trend. However, as the window size increases beyond the critical point, there is also an increase in the possibility of multiple trends in samples being effectively masked into one by correlation and mutual-information calculations causing a reduction in the performance. This trend using the k-NN algorithm is illustrated in Figure 14. Therefore, when using this technique, one needs to be cognizant of the window size and tune it to obtain optimal performance.

As shown in Figure 14, when using the k-NN algorithm with the ISOT AgeTech dataset, a window size of 25 provides optimal performance.

## 7. Conclusions and Future Work

In this research, we successfully demonstrated that some co-located sensors in an IoT smart home maintain linear or nonlinear relationships with one another. We then used a sliding window of size *S* as the contextual attribute under which computations are made for the behavioural attributes, which are the correlation and mutual-information scores among the sensors. These attributes were then successfully used for context-based anomaly detection, achieving high accuracy, decent TPR and low FPR rates. We achieved an accuracy of 96.61%, TPR of 89.47%, FPR of 0% and an AUC of 94.73% with the ISOT AgeTech dataset using a window size of 25 samples.

Because we are modelling relationships between sensors using statistical quantities such as correlation and mutual-information scores, it becomes easier for machine-learning classification models to learn and detect any observation that deviates from this relationship, even when dealing with imbalanced distributions of benign and anomalous data. However, our method can only identify anomalies that violate the existing relationships between sensors. This means that intentional or unintentional anomalies that do not violate the relationship between the sensors cannot be detected.

Additionally, in this study, we utilized a limited number of sensors, including temperature, humidity and air-quality sensors, to build our detection model. However, as we intend for future work, it would be beneficial to include more sensors, such as luminance sensors to detect ambient lighting, PIR sensors to detect motion, moisture sensors to detect incontinence [37], smart scales to measure weight [38], fall-detection sensors to detect falls [39] and wearable sensors to detect heart rate, sleep and steps taken. This would result in more relationships, which could make our technique more robust.

## Figures and Tables

**Table 1:** Examples of commonly used AgeTech sensors.

IoT Device	Protocol	Role in AgeTech	Potential Implementation
IP Cam	WiFi	Surveillance	ESP32-CAM with ESP32 MC
Thermostat	WiFi/Bluetooth	Temperature Control	DHT22 with ESP32 MC
Flame Detector	WiFi/Bluetooth	Fire Protection	MQ135, KY-026, with ESP32 MC.
Smart Light	WiFi/Bluetooth	Illumination	BH1750 with ESP32 MC
Smart Lock	WiFi/Bluetooth	Security	ZFM-20, Servo motor, with ESP32 MC
SPO2, Heart Rate Monitor	Bluetooth	Monitoring Vitals	MAX30101 with ESP32 MC
Fall Detection Sensors	Bluetooth/Others	Fall Detection	MEMS - MPU6050 with ESP32 MC
Smart Pillbox	WiFi/Bluetooth	Medicine Administration	Chassis, Servo motors and ESP32 MC
Smart Scale	WiFi/Bluetooth	Weight Measurement	FC2231, HX711 with ESP32
Smart Diaper	Bluetooth	Incontinence Measurement	SEN-13322 with ESP32 MC

**Table 2:** Placement of the sensors in different rooms for the open smart-home dataset.

Location	Brightness	Humidity	Temperature	Thermostat
Bathroom	1	1	1	1
Kitchen	1	1	1	1
Room 1	1	1	1	1
Room 2	1	1	1	1
Room 3	1	1	1	2
Toilet	1	1	1	1

**Table 3:** Number of data samples per sensor type in the open smart-home dataset.

Type	Counts of Samples
Brightness	64,629
Humidity	10,076
Temperature	81,029
Thermostat Temp.	74,046

**Table 4:** Distribution of data samples per sensor type in the smart-building dataset.

Sensor Type	Counts of Samples
Luminance	6,571,412
Temperature	6,571,454
CO_2_	6,573,957
PIR	3,593,902
Humidity	6,571,414

**Table 5:** Descriptions of DHT22 and MQ135 sensors.

Sensor	Description
DHT22	The DHT22 is a low-cost digital temperature and humidity sensor.
MQ135	MQ135 is an air-quality sensor that is extremely sensitive to benzene, sulfide, smoke and other harmful gases.

**Table 6:** Examples of temperature and humidity sensor (3R32) data collected in the ISOT AgeTech dataset.

Timestamp	Temperature	Humidity	Type
1662689838001	44.09999851	20.29999924	normal
1662689868083	44.09999843	20.29999922	normal
1662689898161	44.09999847	20.29999926	normal
1662689928240	44.09999847	20.29999923	normal
1662689958321	44.20000076	20.20000036	normal

**Table 7:** First five rows of the merged ISOT AgeTech dataset.

Time	Temp. (3R32)	Hum. (3R32)	Temp. (3U38)	Hum. (3U38)	AirQ. (3U46)	AirQ. (3U48)	Type
1662274800	49.09	21.0	49.70	20.70	290	238	benign
1662274830	49.09	21.0	49.70	20.79	291	243	benign
1662274860	49.20	20.9	49.70	20.70	290	242	benign
1662274890	49.20	20.9	49.78	20.70	289	244	benign
1662274920	49.20	20.9	49.77	20.70	273	245	benign

**Table 8:** Distribution of benign and anomalous samples in the merged ISOT AgeTech dataset.

Type	Number of Samples
benign	8550
intentional anomalies	2190
unintentional anomalies	418

**Table 9:** Interpretation of Pearson’s correlation coefficient.

Range	Level	Range	Level
0.8 to 1.0	Very Strong Positive	−1.0 to −0.8	Very Strong Negative
0.6 to 0.79	Strong Positive	−0.79 to −0.60	Strong Negative
0.4 to 0.59	Moderate Positive	−1.0 to −0.8	Moderate Negative
0.2 to 0.39	Weak Positive	−0.39 to −0.20	Weak Negative
0.00 to 0.19	Very Weak Positive	−0.19 to −0.01	Very Weak Negative

**Table 10:** Detection performance using a window of size 15 on the transformed ISOT AgeTech dataset.

Classifier	Accuracy	TPR	FPR	AUC
Random Forest	85.15%	59.45%	0%	79.72%
k-NN	85.15%	65.57%	4.68%	81.44%
Naive Bayes	80.19%	70.27%	14.06%	78.11%

**Table 11:** Detection performance using a window of size 25 on the transformed ISOT AgeTech dataset.

Classifier	Accuracy	TPR	FPR	AUC
Random Forest	94.91%	84.21%	0%	92.10%
k-NN	96.61%	89.47%	0%	94.73%
Naive Bayes	72.88%	36.84%	10%	63.42%

**Table 12:** Detection performance with a window of size 35 on the transformed ISOT AgeTech dataset.

Classifier	Accuracy	TPR	FPR	AUC
Random Forest	87.50%	65.0%	0%	82.50%
k-NN	89.28%	70.0%	0%	85.0%
Naive Bayes	67.85%	20.0%	5.55%	57.22%

**Table 13:** Results from applying traditional deep-learning approaches on ISOT AgeTech dataset.

Classifier	Accuracy	TPR	FPR	AUC
LSTM	91.62%	72.45%	2.24%	85.10%
Simple RNN	96.81%	86.80%	0%	93.40%

## Data Availability

The dataset, models and code used during the study are available on request.
